# F212. IMPROVEMENT IN COGNITIVE BIASES AFTER GROUP PSYCHOEDUCATION AND METACOGNITIVE TRAINING IN RECENT ONSET PSYCHOSIS

**DOI:** 10.1093/schbul/sby017.743

**Published:** 2018-04-01

**Authors:** Maria Isabel Ahuir Perez, Ángel Cabezas, Maria José Miñano, Maria José Algora, Francesc Estrada, Montserrat Sole, Juan David Barbero, Itziar Montalvo, Alfonso Gutierrez-Zotes, Vanessa Sanchez-Gistau, José Antonio Monreal, Elisabet Vilella, Diego Palao, Javier Labad

**Affiliations:** 1 Parc Taulí Hospital Universitari, I3PT, Universitat Autònoma de Barcelona; 2 Early Intervention Service, Hospital Universitari Institut Pere Mata, IISPV, Universitat Rovira i Virgili; 3 Hospital Universitari Institut Pere Mata, IISPV, Universitat Rovira i Virgili; 4 Hospital Univeristari institut Pere Mata, IISP, Universitat Rovira i Virgili

## Abstract

**Background:**

Group psychotherapeutic treatments can improve negative symptoms and social functioning deficits in the treatment of schizophrenia. These treatments may include different modalities including group cognitive behavioral therapy, psychoeducation and metacognitive training (MCT). MCT is effective for preventing delusions by modifying the cognitive biases most related to psychosis. Our primary goal was to address whether cognitive biases improve more specifically with MCT when compared to psychoeducation in a sample of patients with recent onset psychosis.

**Methods:**

Design: a multicenter randomized, pilot clinical trial was performed, in which one group received psychoeducation and the other MCT.

**Sample:**

49 patients aged between 18–35 years and with a diagnosis of psychotic disorder according to DSM-IV-TR criteria and less than 3 years of duration of illness. All patients were recruited at two Early Psychosis Programmes in Spain (ParcTaulí Hospital Universitari, Sabadell; Hospital UniversitariInstitut Pere Mata, Reus). Ethical approval was obtained from the local Ethics Committees of both institutions.

**Outcomes:**

Patients were evaluated at baseline and at the end of each intervention. The primary outcome was cognitive biases, assessed with Cognitive Biases Questionnaire for Psychosis (CBQ). Secondary outcomes included cognitive insight, psychopathological symptoms (positive, negative, depressive) and psychosocial functioning.

**Interventions:**

The interventions consisted of 8 weekly group sessions of MCT (developed at the University of Hamburg-Eppendorf by Steffen Moritz) or psychoeducation. MCT program included sessions dealing with attributional style, jumping to conclusions, changing beliefs, empathy, memory, and depression and self-esteem. The psychoeducational program included sessions addressing aspects related to psychotic illness (psychotic symptoms, risk factors of relapse, stress management, psychopharmacological treatment, substance use, physical health and social skills).

**Statistical analysis:** A general linear model for repeated measures was performed in order to compare the longitudinal effect of the intervention and to test whether changes in outcome variables differed by treatment group. All analyses were adjusted for gender. A p value < 0.05 (two-tailed) was considered to be significant.

**Results:**

Of all 49 patients, 38 (77.6%) completed at least 50% of the sessions, and were included in the final analyses. 21 received psychoeducation and 16 MCT.

Cognitive biases improved significantly in both psychoeducation (43.8 ± 11.2 vs 40.8 ± 10.4) and MCT groups (44.2 ± 7.6 vs 39.6 ± 5.0). The time effect was significant (F= 18.9, p<0.001) without a different pattern in the change of CBQ scores between groups (interaction time x group, F= 0.63, p= 0.431). An improvement in negative symptoms was also observed after receiving both treatments, without significant differences between groups. No significant changes over time were observed in positive symptoms, depressive symptoms or psychosocial functioning.

**Discussion:**

Both group psychoeducation and MCT improve cognitive biases in recent onset psychosis. Our study does not support a superiority of one intervention over the other in terms of improving cognitive biases.

